# Supplementary material for: Competence remodels the pneumococcal cell wall exposing key surface virulence factors that mediate increased host adherence
Source: PLoS Biol. 2023 Jan 30;21(1):e3001990. doi: 10.1371/journal.pbio.3001990 (PMC9910801; doi:10.1371/journal.pbio.3001990)
Supplement: S1 Fig — (A, B) Variance of the replicates. (C) IPTG main effect in competence by interaction of p-values. Tested against fold change of 2 (green lines) with alpha of 0.05 (red line) (raw data in S1 Table). (DOCX) [file pbio.3001990.s001.docx]

**
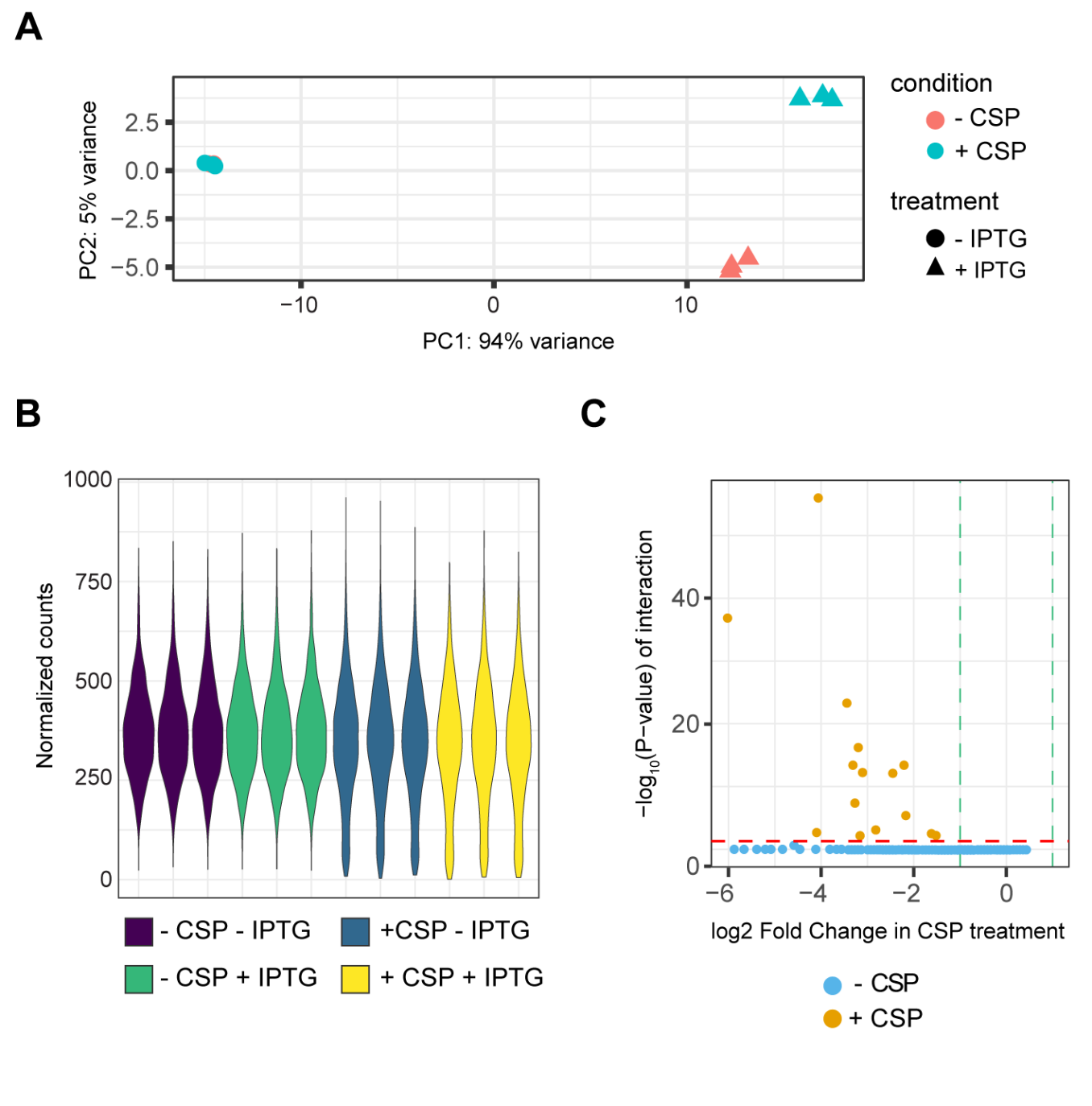
**

**S1 Fig. Evaluation of fitness cost during competence using CRISPRi pool screen. A-B**) Variance of the replicates. **C**) IPTG main effect in competence by interaction of p−values. Tested against fold change of 2 (green lines) with alpha of 0.05 (red line) (raw data in S1 Table).
