# Supplementary material for: Competence remodels the pneumococcal cell wall exposing key surface virulence factors that mediate increased host adherence
Source: PLoS Biol. 2023 Jan 30;21(1):e3001990. doi: 10.1371/journal.pbio.3001990 (PMC9910801; doi:10.1371/journal.pbio.3001990)
Supplement: S2 Fig — (A) sgRNAs with a significant fitness cost during competence. (B) Other sgRNAs related to competence or TA synthesis with no fitness cost. Fitness cost was evaluated as described before (de Bakker and colleagues) [53] (see Methods for more details) (raw data in S1 Table). (DOCX) [file pbio.3001990.s002.docx]

**
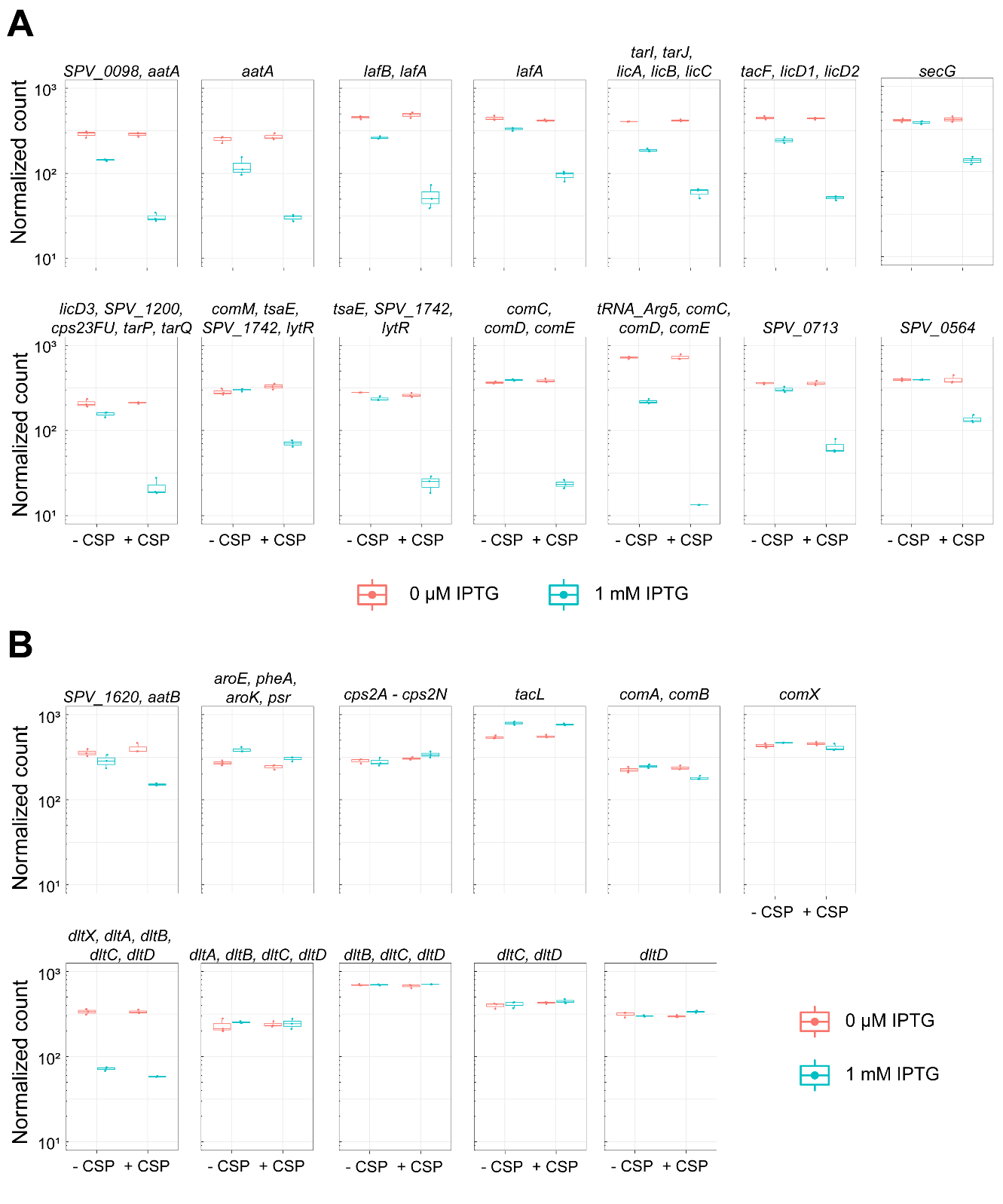
**

**S2 Fig. Normalized counts of sgRNAs related to competence and teichoic acid (TA) synthesis. A)** sgRNAs with a significant fitness cost during competence**. B)** other sgRNAs related to competence or TA synthesis with no fitness cost. Fitness cost was evaluated as described before (de Bakker et al., 2022) (see methods for more details) (raw data in S1 Table).
