# Supplementary figures and images for: Competence remodels the pneumococcal cell wall exposing key surface virulence factors that mediate increased host adherence
Source: PLoS Biol. 2023 Jan 30;21(1):e3001990. doi: 10.1371/journal.pbio.3001990 (PMC9910801; doi:10.1371/journal.pbio.3001990)

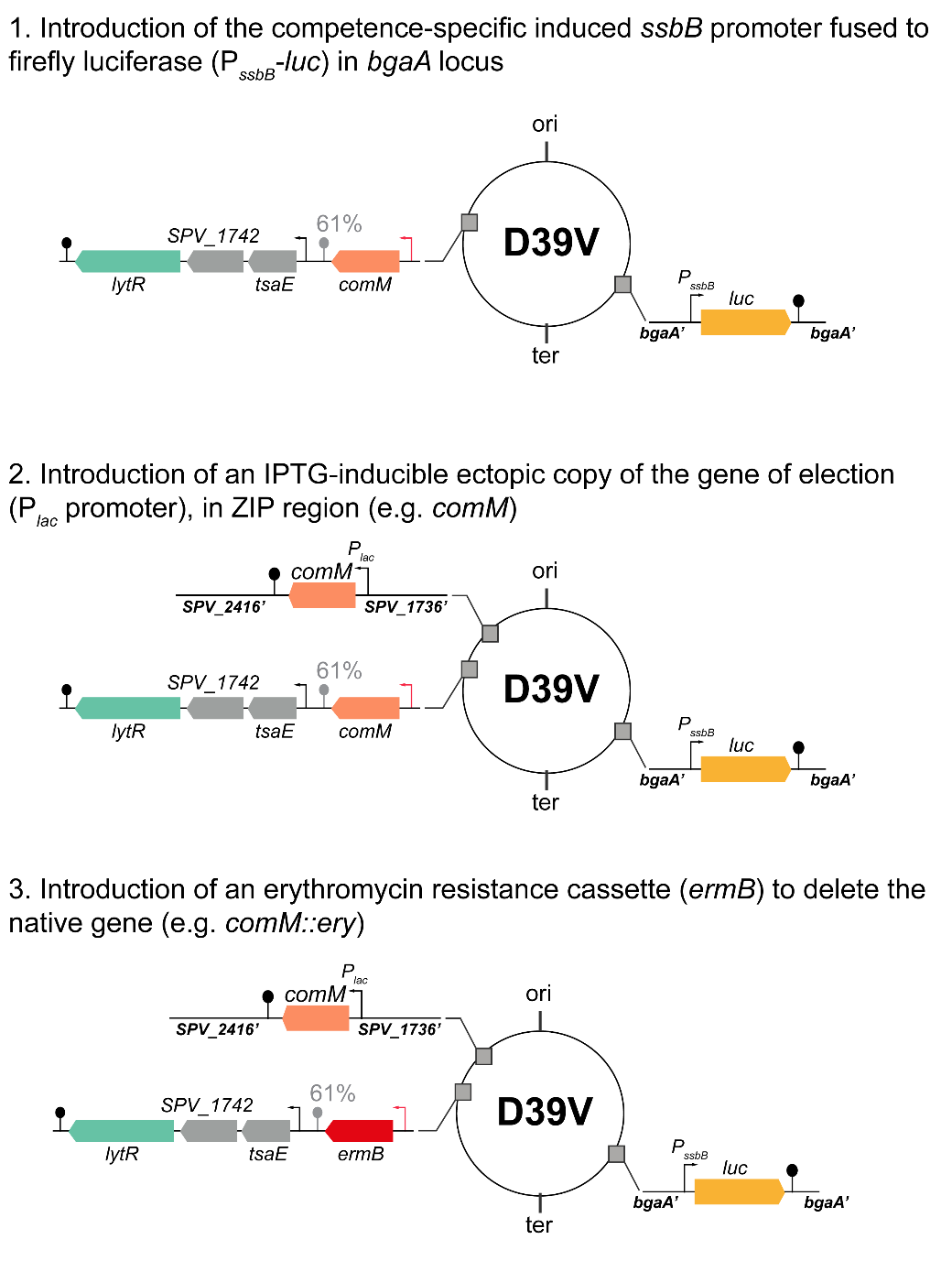


**S3 Fig.** Design of inducible systems.

Supplement: S3 Fig — (DOCX) [file pbio.3001990.s003.docx]
