# Supplementary material for: Competence remodels the pneumococcal cell wall exposing key surface virulence factors that mediate increased host adherence
Source: PLoS Biol. 2023 Jan 30;21(1):e3001990. doi: 10.1371/journal.pbio.3001990 (PMC9910801; doi:10.1371/journal.pbio.3001990)
Supplement: S4 Fig — Phase contrast, DAPI staining, and Nile red staining are displayed. (DOCX) [file pbio.3001990.s004.docx]

**
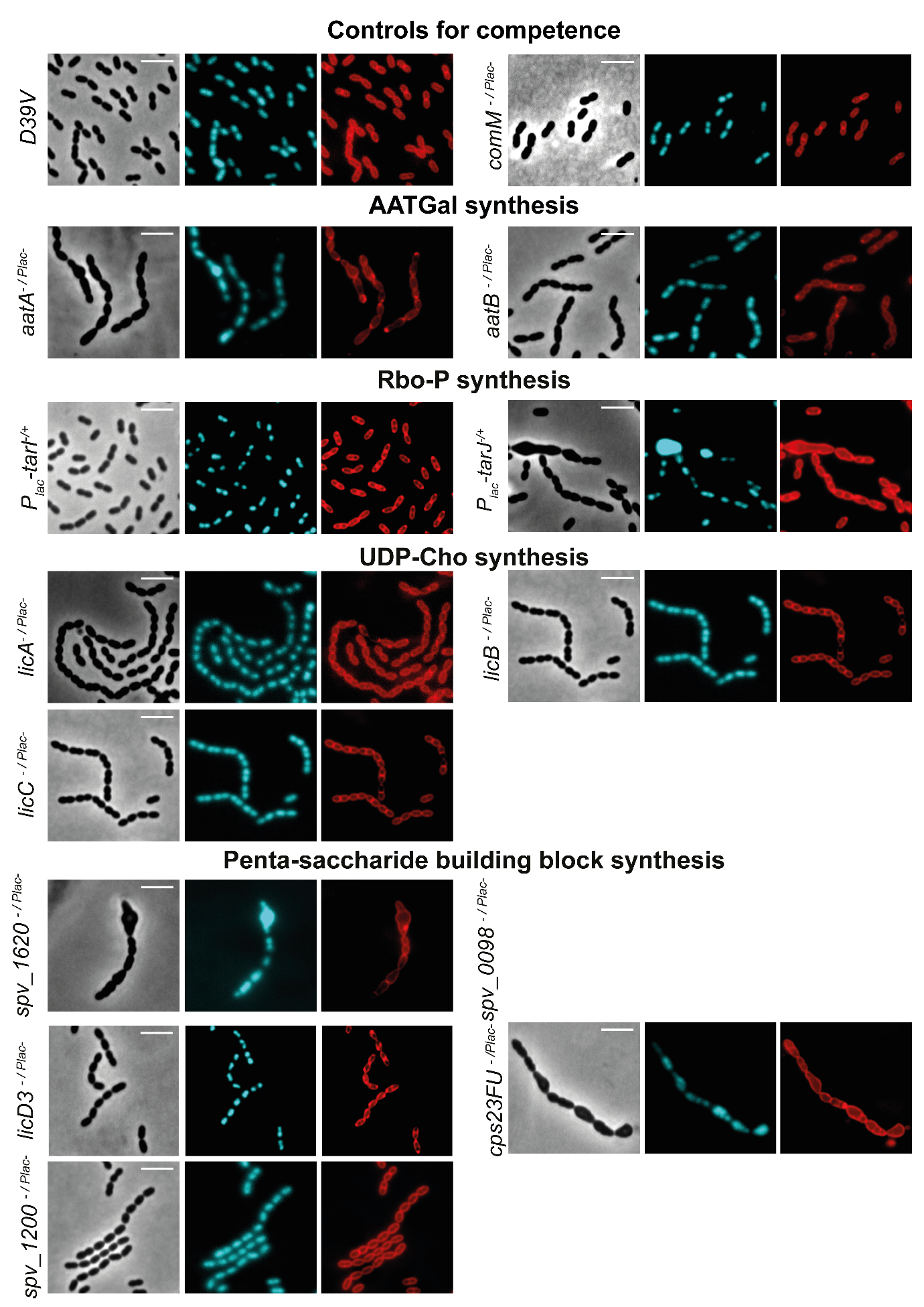

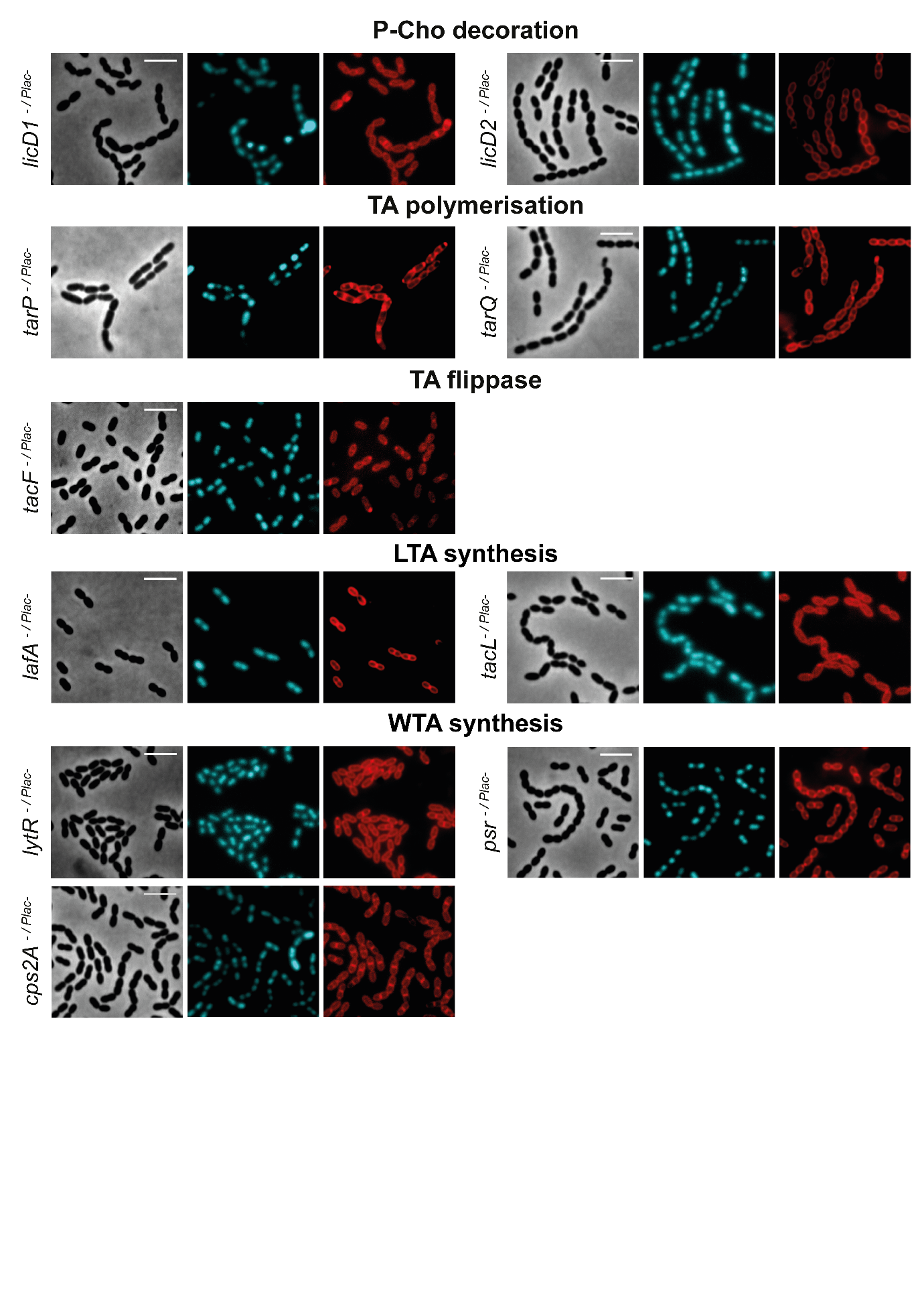
**

**S4 Fig.** Morphological changes were examined with fluorescence microscopy, and representative micrographs are shown. Phase contrast, DAPI staining, and Nile red staining are displayed.
