# Supplementary material for: Competence remodels the pneumococcal cell wall exposing key surface virulence factors that mediate increased host adherence
Source: PLoS Biol. 2023 Jan 30;21(1):e3001990. doi: 10.1371/journal.pbio.3001990 (PMC9910801; doi:10.1371/journal.pbio.3001990)
Supplement: S5 Fig — Every strain contains a depletion system by ectopically expressing the indicated gene under control of the Plac IPTG-inducible promoter and the deletion of the gene from its native location. Top, growth curves in absence of the protein (blue = No IPTG) or with different IPTG concentrations. Bottom, area under the curve of relative luminescence values (RLU) of a PssbB-luc construct. For typical PssbB-luc profiles, see, e.g., Slager and colleagues [56]. Note that in absence of comCDE, no signal is visible in this kind of data visualization (e.g., see panel 2 Fig 1A, NO IPTG) (raw data in S9 Table). (DOCX) [file pbio.3001990.s005.docx]

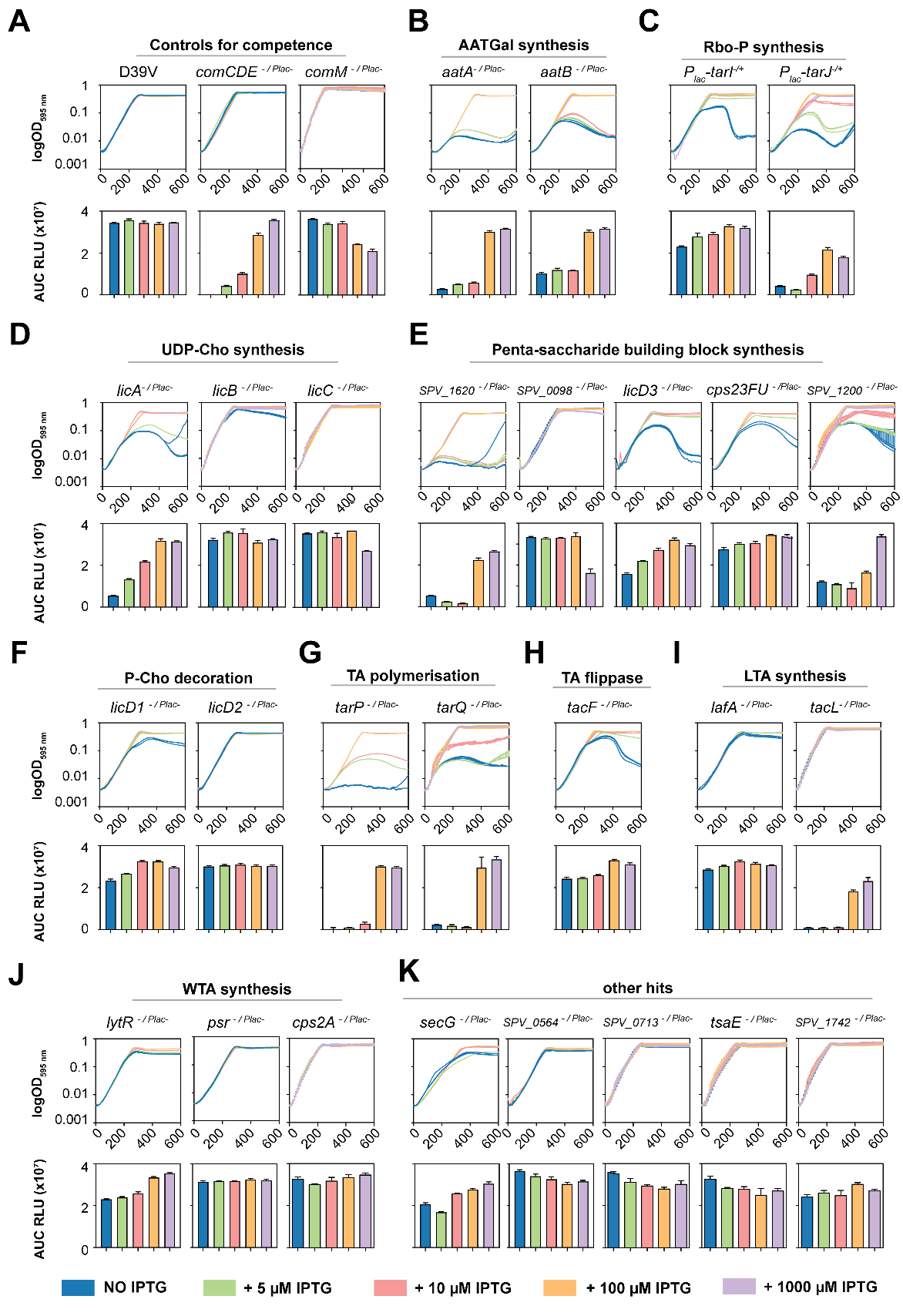


**S5 Fig. Natural competence development in strains depleted for competence and teichoic acid related genes.** Every strain contains a depletion system by ectopically expressing the indicated gene under control of the Plac IPTG-inducible promoter, and the deletion of the gene from its native location. Top, growth curves in absence of the protein (blue = No IPTG) or with different IPTG concentrations. Bottom, area under the curve (AUC) of relative luminescence values (RLU) of a *PssbB-luc* construct**.** For typical *PssbB-luc* profiles, see e.g. Slager et al., 2014 *Cell* and Prudhomme et al., 2006 *Science*. Note that in absence of comCDE, no signal is visible in this kind of data visualization (e.g. see panel 2 Fig. 1A, NO IPTG) (raw data in S9 Table).
