# Supplementary material for: Competence remodels the pneumococcal cell wall exposing key surface virulence factors that mediate increased host adherence
Source: PLoS Biol. 2023 Jan 30;21(1):e3001990. doi: 10.1371/journal.pbio.3001990 (PMC9910801; doi:10.1371/journal.pbio.3001990)
Supplement: S6 Fig — Individual strains were grown in C+Y pH 6.8 to avoid natural competence development in presence of SYTOX Green Dead Cell Stain dye. When OD595 nm reached approximately 0.1, 100 ng/ml of CSP1 was added to induce competence. For those genes showing less cell lysis in our setup, experiment was performed in absence of IPTG (green and orange). For those essential with severe growth defect, 10 μM IPTG was added to maintain a mild expression of the protein and avoid cell lysis in absence of CSP due to the essentiality of the gene (blue and pink) (raw data in S10 Table). (DOCX) [file pbio.3001990.s006.docx]

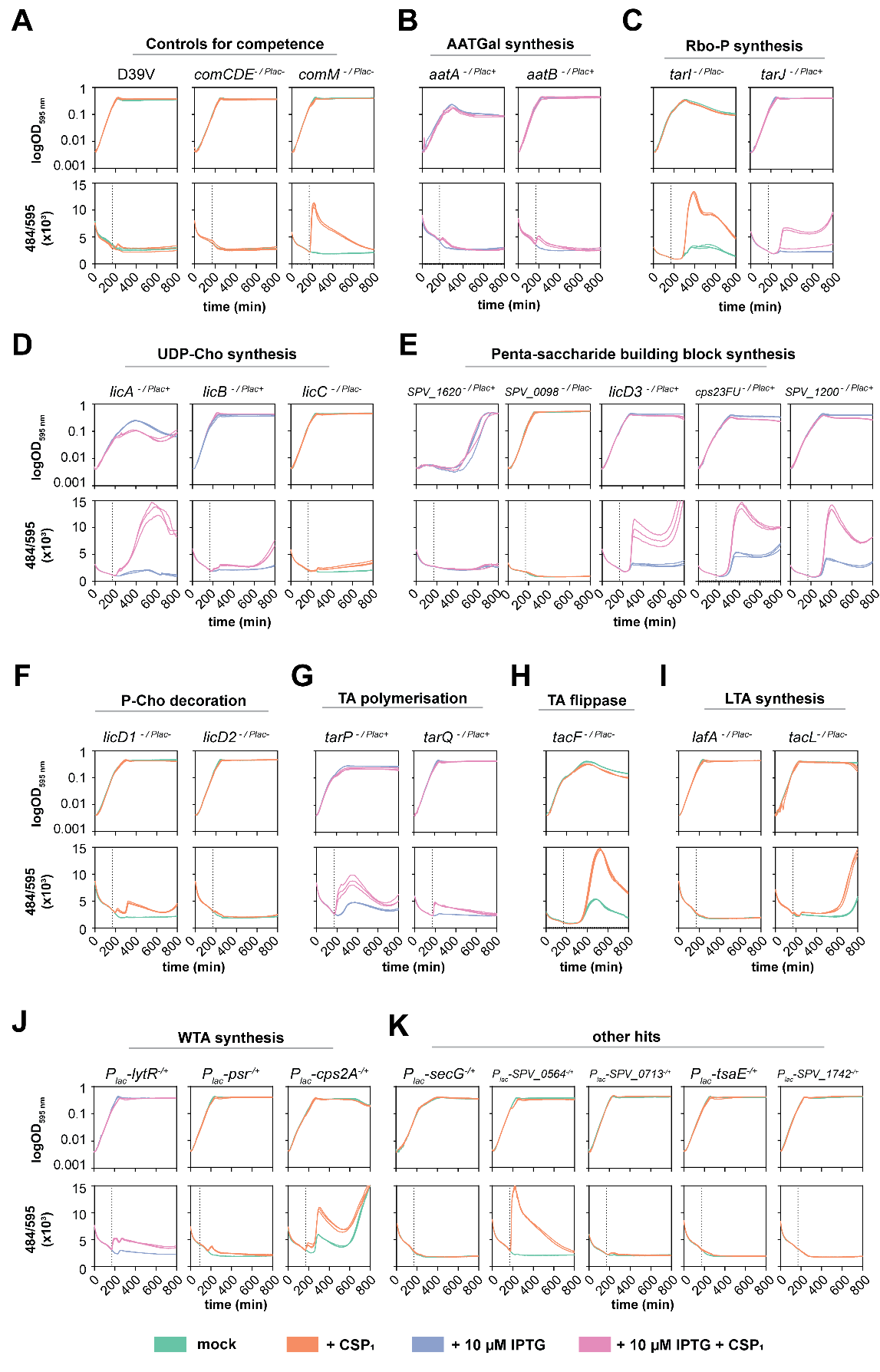


**S6 Fig. Detection of cell lysis.** Individual strains were grown in C+Y pH 6.8 to avoid natural competence development in presence of SYTOX Green Dead Cell Stain dye. When OD_595 nm_ reached ~ 0.1, 100 ng/ml of CSP_1_ was added to induce competence. For those genes showing less cell lysis in our setup, experiment was performed in absence of IPTG (green and orange). For those essential with severe growth defect, 10 µM IPTG was added to maintain a mild expression of the protein and avoid cell lysis in absence of CSP due to the essentiality of the gene (blue and pink) (raw data in S10 Table).
