# Supplementary material for: Competence remodels the pneumococcal cell wall exposing key surface virulence factors that mediate increased host adherence
Source: PLoS Biol. 2023 Jan 30;21(1):e3001990. doi: 10.1371/journal.pbio.3001990 (PMC9910801; doi:10.1371/journal.pbio.3001990)
Supplement: S7 Fig — (A) D39V cells were grown to OD 0.1 in C+Y medium pH 7.4, then exposed to 100 ng/ml or 0 ng/ml of CSP1 for 30 min. Samples were subjected to epifluorescence microscopy, where phase contrast images were used to measure cell length. Diamond symbols represent outlier individual cell length measurements. Asterisks show statistically significant differences in cell length (Mann–Whitney U test) (see Methods section for more details). (B) D39V strains containing aTc inducible promoters coupled to cbpD or comM were grown to OD 0.1 in C+Y medium pH 7.4, then exposed to 100 ng/ml or 0 ng/ml of CSP1 for 30 min and/or 100 ng/ml aTc. Cells were then stained with primary antibodies raised against PspA and then Goat anti-rabbit IgG (H+L) Alexa 555, both at 1/500 dilutions and subjected to epifluorescence microscopy. Fluorescence intensity based on phase-contrast and fluorescence composite images was measured. Diamond symbols represent outlier individual fluorescence measurements. Asterisks show statistically significant differences in cell length (Mann–Whitney U test) (see Methods section for more details). (C) Representative phase contrast and fluorescence composite image taken of D39V strain with comM genetically tagged with YFP at its C-terminus. (D) D39V Δcps cells were grown to OD 0.1 in C+Y medium pH 7.4, then exposed to 100 ng/ml (left image) or 0 ng/ml (right image) of CSP1 for 30 min. Cells were then stained with primary antibodies raised against PspA, and then Goat anti-rabbit IgG (H+L) Alexa 555, both at 1/500 dilutions and subjected to epifluorescence microscopy (see Methods section for more details). (E) Adherence assay. D39V (WT and ΔcbpD) strains were inoculated with lung epithelial A549 cells to a multiplicity of infection of approximately 20 in RPMI 1640 without phenol red supplemented with 1% (v/v) FBS and 10 mM HEPES buffer for 2 h at 37 °C. Cells were then detached, and appropriate dilutions of the cultures were plated on blood agar plates to determine the numb [file pbio.3001990.s007.docx]

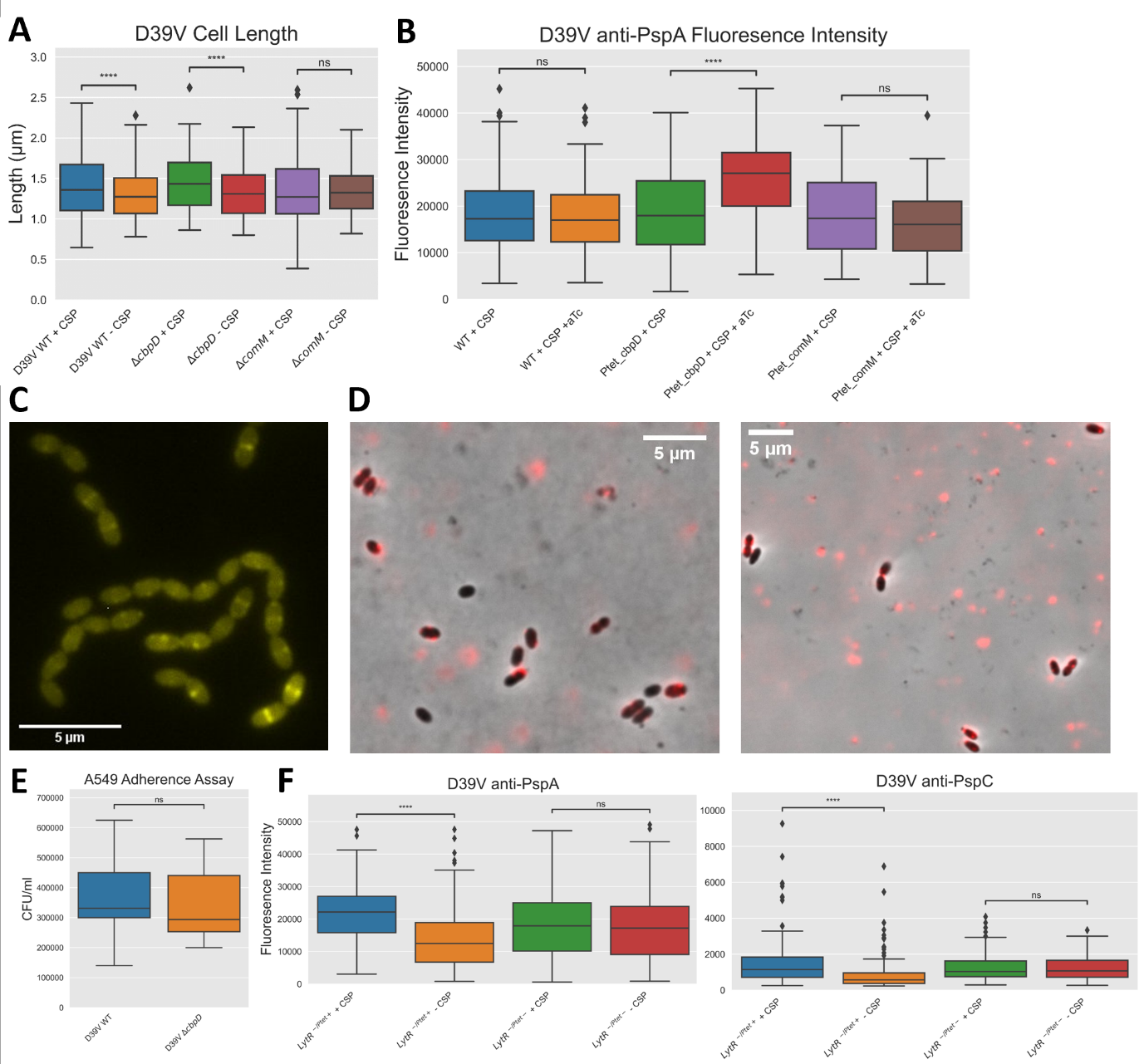
 **S7 Fig. Cell length measurements and PspA overexpression immunofluorescence after competence induction, PspA immunofluorescence of a ∆*cps* mutant, adherence assay and PspA and PspC immunofluorescence with LytR overexpression or depletion. A**) D39V cells were grown to OD 0.1 in C+Y medium pH 7.4, then exposed to 100 ng/ml or 0 ng/ml of CSP-1 for 30 min. Samples were subjected to epifluorescence microscopy, where phase contrast images were used to measure cell length. Diamond symbols represent outlier individual cell length measurements. Asterisks show statistically significant differences in cell length (Mann-Whitney U test) (see methods section for more details). **B**) D39V strains containing aTc inducible promoters coupled to *cbpD* or *comM* were grown to OD 0.1 in C+Y medium pH 7.4, then exposed to 100 ng/ml or 0 ng/ml of CSP-1 for 30 min and/or 100ng/ml aTc**.** Cells were then stained with primary antibodies raised against PspA and then Goat anti-rabbit IgG (H+L) Alexa 555, both at 1/500 dilutions and subjected to epifluorescence microscopy. Fluorescence intensity based on phase-contrast and fluorescence composite images was measured. Diamond symbols represent outlier individual fluorescence measurements. Asterisks show statistically significant differences in cell length (Mann-Whitney U test) (see methods section for more details). **C**) Representative phase contrast and fluorescence composite image taken of D39V strain with *comM* genetically tagged with YFP at its C-terminus. **D**) D39V Δ*cps* cells were grown to OD 0.1 in C+Y medium pH 7.4, then exposed to 100 ng/ml (left image) or 0 ng/ml (right image) of CSP-1 for 30 min. Cells were then stained with primary antibodies raised against PspA, and then Goat anti-rabbit IgG (H+L) Alexa 555, both at 1/500 dilutions and subjected to epifluorescence microscopy (see methods section for more details). **E**) Adherence assay. D39V (WT and Δ*cbpD*) strains were inoculated with lung epithelial A549 cells to a multiplicity of infection of ~20 in RPMI 1640 without phenol red supplemented with 1% (v/v) FBS and 10mM HEPES buffer for 2 h at 37^o^C. Cells were then detached, and appropriate dilutions of the cultures were plated on blood agar plates to determine the number of adherent bacteria (see methods section for more details. Data presented are the means ± standard deviation (ns, not-significant, unpaired t-test). **F**) D39V (LytR ^-/Ptet-^ and LytR ^-/Ptet+^) were grown to OD 0.1 in C+Y medium pH 6.9, then exposed to 100 ng/ml or 0 ng/ml of CSP1 for 30 min as well as 0μM or 100μM IPTG. Strains containing a complementing copy are indicated by Plac (- indicates no IPTG, + indicates addition of 100 μM IPTG). Cells were then stained with primary antibodies raised against PspA or PspC, and then Goat anti-rabbit IgG (H+L) Alexa 555, both at 1/500 dilutions and subjected to epifluorescence microscopy. Fluorescence intensity based on phase-contrast and fluorescence composite images were measured. Diamond symbols represent outlier individual cells. Asterisks show statistically significant differences in fluorescence intensity (ns, not-significant and ****, *P <*0.0001, Mann-Whitney U test) (see methods section for more details) (raw data in S5 and S6 Tables).
