# Supplementary material for: Competence remodels the pneumococcal cell wall exposing key surface virulence factors that mediate increased host adherence
Source: PLoS Biol. 2023 Jan 30;21(1):e3001990. doi: 10.1371/journal.pbio.3001990 (PMC9910801; doi:10.1371/journal.pbio.3001990)
Supplement: S8 Fig — (A) Cell lysis detection in D39V, ΔcomM and double ΔcomMΔcbpD strains. Individual strains were grown in C+Y medium (top) or C medium (bottom) at pH 6.8 to avoid natural competence development in presence of SYTOX Green Dead Cell Stain dye. When cell cultures reached OD595 nm 0.1 (approximately after 170 min), 100 ng/ml of CSP1 was added to induce competence (orange lines). Three biological replicates per condition are shown. (B) Evaluation of cell lysis in the strains used for the radioactive assay (Fig 4). Cells were grown in the indicated medium in presence of SYTOX Green Dead Cell Stain dye. Three biological replicates per condition are shown (raw data in S11 Table). (DOCX) [file pbio.3001990.s008.docx]

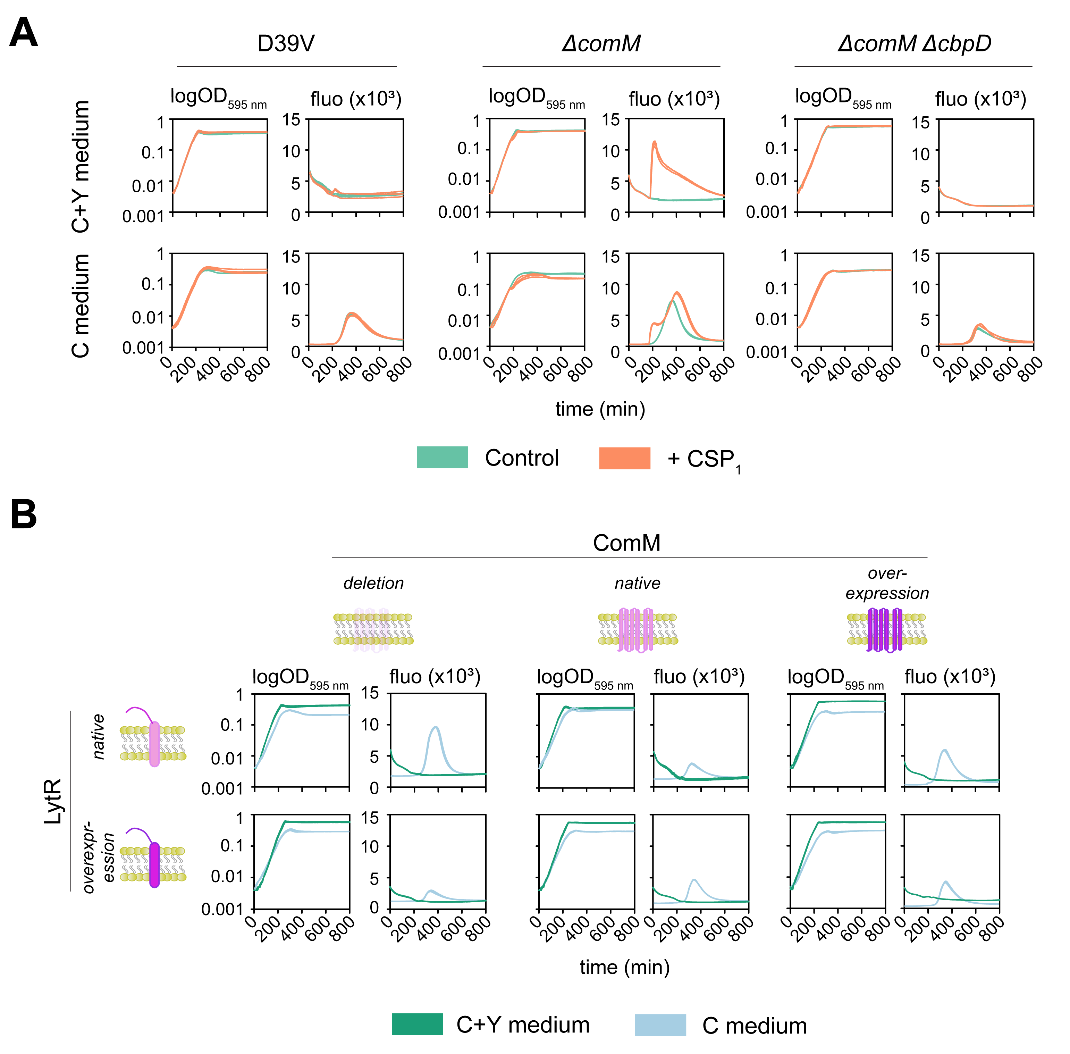


**S8 Fig. Cell lysis detection in C+Y and C media. A)** Cell lysis detection in D39V, Δ*comM* and double Δ*comM*Δ*cbpD* strains. Individual strains were grown in C+Y medium (top) or C medium (bottom) at pH 6.8 to avoid natural competence development in presence of SYTOX Green Dead Cell Stain dye. When cell cultures reached OD_595 nm_ 0.1 (~ after 170 min), 100 ng/ml of CSP_1_ was added to induce competence (orange lines). Three biological replicates per condition are shown. **B)** Evaluation of cell lysis in the strains used for the radioactive assay (Fig 4). Cells were grown in the indicated medium in presence of SYTOX Green Dead Cell Stain dye. Three biological replicates per condition are shown (raw data in S11 Table).
