# Supplementary material for: Competence remodels the pneumococcal cell wall exposing key surface virulence factors that mediate increased host adherence
Source: PLoS Biol. 2023 Jan 30;21(1):e3001990. doi: 10.1371/journal.pbio.3001990 (PMC9910801; doi:10.1371/journal.pbio.3001990)
Supplement: Attachment — Submitted filename: Response_to_reviewers.pdf [file pbio.3001990.s020.pdf]

We would like to thank the editors and reviewers for taking the time to go through our manuscript and provide advice that has greatly improved the quality of this study. You will find below a point-by-point response to each comment raised by the reviewers, including any changes to the manuscript that were made. In particular, we have addressed the comments raised by reviewer 3 on the 2nd section of the manuscript. We have better linked adherence to host cells to the exposure of choline binding proteins (CBPs) on the bacterial surface. This has been done through the addition of new experiments that look at LytR depletion on CBP surface exposure and adherence. These new results provide additional evidence demonstrating that TA synthesis impacts deposition of CBPs on the cell surface, and this impacts pneumococcal ability to adhere to nasopharyngeal cells. Moreover, we have included additional references into the MS that further highlight the impact of CbpD and CBPs in general on pneumococcal virulence.

Reviewer 1:

1) *Line 82: The original reference for choline binding proteins attaching to Pcho on teichoic acids is not Galan 2015. Suggestions are…:*

   Citations have been adjusted to the suggested papers

2) *The difference in apparent distribution of Cbps when measured by antibody binding vs GFP is noteworthy. At first it could be potentially explained by increased access of antibody to the growth zone perhaps due to loss of capsule. However, this would not agree with older work from the Briles lab where capsule does not block PspA antibody but does block antiphosphorylcholine antibodies. Please discuss:*

   The contradiction between capsule shedding increasing antibody binding to PspA we propose, to the Briles lab paper (Daniels et al, 2006) which showed capsule does not impact masking of PspA to mono-clonal antibodies, may be due to the different media the strains were grown in, as capsule amount and hence ability to mask PspA, would be altered. In fact, the Daniels paper does show slightly better binding of PspA polyclonal antibodies in an unencapsulated D39 vs wild type capsulated bacteria. Unfortunately, the Daniels et al paper does not provide which media they grew their pneumococcal strains in, so this point cannot be tested. In addition, our data are not contrary to the Daniels et al study as PspA might be overall surface exposed, but we show it is much more surface-exposed at midcell during competence. It could also be that the antibody raised against PspA in our study is reactive to different PspA epitopes than the antibody raised in the Daniels study (i.e. higher affinity to domains in the protein closer to the inner membrane). The sera from the Daniels et al study was first reported in Vela Coral, Emerging Infectious Diseases 2001, while our polyclonal antibody was produced in Hammerschmidt et al. Infection & Immunity 1999. This apparent discrepancy has now been discussed in the revised manuscript. In addition, we re-drew our model (Figure 6) to also show some surface exposure of PspA in non-competent cells and changed the legend so it is consistent with the observations made previously.

3) *On occasion it is not clear what statements are based on literature vs representing a hypothesis that would explain current results that are not directly shown.*

   a: Line 370/483: As the experiments are presented statements are made that CbpD results in capsule shedding. However, this is not shown experimentally and thus should be hypothesized. Similarly, CbpD hydrolyzes cell wall (details unclear), and it is not known if this results in release of cell wall components:

   Text has been adjusted to clarify that we are hypothesizing upon the results

   b: Please clarify the literature evidence that Cbps attach preferentially to WTA vs LTA? Or modify language to suggest this as a hypothesis supported by your work:

   We do not state in the paper that CBPs attach preferentially to either WTA or LTA. Only that the differential expression of LytR, ComM and TacL leads to differing ratios of WTA/LTA amounts in the cell wall.

   c: Please clarify if there is literature evidence that LytR attaches TA to cell wall in addition to the results that are compatible with but do not directly show it in this paper:

   We have now cited a study that showed that deletion of *lytR* led to decreased retention of TAs to the pneumococcal surface (Ye et al. 2018).

Reviewer 2:

1) *2. Reverse "surface exposing":*

   We have rephrased the title to help clarify title meaning to: Competence remodels the pneumococcal cell wall providing resistance to fratricide and exposes key surface virulence factors

2) *107. Why is this most likely?:*

   We agree with the referee and have rephrased this. Eldholm et al., 2010 indeed show that CbpD has muralytic activity, and that this activity requires functional CBD and SH3b domains.

3) *128. 'here we report':*

   Wording has been adjusted accordingly

4) *137. Shouldn't "to" be "on"?:*

'to' has been switched to 'on' to better clarify the meaning of the sentence

5) *163. This may be just a matter of taste but: the use of "essential" in this context may be misleading. We usually consider that if something essential is missing, the cell will be dead. Unless I misread the results, what is reported/detected here is that cells missing some identified genes are more susceptible to lytic killing, not absolutely "dead in the water.":*

   The sentence has been edited to clarify that essential is meant in terms of more essential in protecting cells against lysis during competence

6) *164. Please define "top".*

   Changed 'top' to 'most essential'

7) *169.-175. This text may be too telegraphic. A naïve reader may be confused as to which operons are which:*

   Edited the text to clarify what the first operon is

8) *178. Is this pathway proposed here, or is a citation to its previous proposal in order?:*

   The pathway shown here was constructed on basis of prior knowledge. These references have now been included.

9) *202. 10 TA operons?:*

   10 operons that contain the 14 sgRNAs found to become more essential in protecting cells against lysis during competence. Text has been edited to make this clear

10) *212. Is this repression or simply gene deletion?*

   This is a tricky point as the native genes are deleted and at an ectopic locus we have a copy under IPTG-inducible control. We changed the phrasing earlier from 'deplete' to 'not produce' to avoid potential confusion as well as changed repression to depletion in old line 212.

11) *211 (fig 5) Please explain a bit more how these patterns should be understood. For example, what is the significance of ssbB expression very late into stationary phase? Also, where could a reader look to find the actual kinetics of its expression?:*

   We decided to plot *ssbB* expression as area under the curve instead as kinetic curves so we are able to show many more data points in the same graph. Note that there is no stationary phase *ssbB* expression, the different bars represent different concentrations of inducer that is being used to control the expression of the gene of interest. For instance, if we look at the second plot, *comCDE*. Here *comCDE* is deleted at the native locus and is being expressed from Plac at

the ZIP locus. In the absence of IPTG, *ssbB-luc* is not activated. With 5 uM of IPTG (the green bar) there is slight expression of *ssbB-luc*. At 1 mM of IPTG there is maximal *ssbB-luc* expression. Typical PssbB-luc curves have been published by us and others before, and we now cite this and made our figure legend more clear.

12) 225. Please explain the L/D scale. Would % lysed be a clearer metric?:

In principle, the used Y-axis is similar to a % lysis as suggested by the referee, as we normalize the fluorescence produced by dead cells (484nm emission) over the cell density (595nm). We do this as some depletion strains grow poorly. We have now better clarified this in the figure legends.

13) 392. How can a del-cps strain be encapsulated? Is there a typo here?:

This is a typo, it is meant to say ‘unencapsulated’. Adjusted accordingly

14) 445. Is "subsequently" intended to mean "consequently"?:

Yes, text has been adjusted accordingly

15) 456. As noted above, "essential" may be an oversimplification:

Edited the text in a similar manner as above

16) 458. The operons do not "show an expected cost". Rather, it is interfering with these operons that creates a fitness cost:

Text has been adjusted to clarify this

17) l. 461. "to the contrary, inactivation of the comAB …. :

Text has been adjusted accordingly

18) *463-5. The text seems confused here:*

Agreed and we have removed this section.

19) 503. Perhaps "required" is too strong:

Changed to ‘have an impact’

20) 814. 'as describeed":

Adjusted accordingly

21) *848-853. Is the volume unit "ml" really intended?:*

Many thanks for spotting this mistake. Indeed, it should be micro liter. It has been corrected in the revised manuscript.

22) *855. Note that "to an OD of 0.1" is redundant:*

Removed this from the sentence

23) *857 and ff. Some steps have a temperature indicated, whilst others are not specified:*

Adjusted text to add in missing temperatures

Reviewer 3, specific comments:

1. *Lines 227-228: The authors have shown that ComM and TAs are important to maintain the integrity of cells during competence activation. To show that this process is in fact fratricide mediated, could the authors test whether these genes contribute to lysis in a fratricin-mutant (eg CbpD) background?:*

   In figure 2D and figure S6, these genes were tested for contribution to cell lysis in C + Y pH 6.8 media, which inhibits the development on natural competence and hence, inhibits the expression of *cbpD*.

2. *Lines 238-241: The authors suggests that genes encoding for other LCP family proteins - cps2A and psr are not important for TA attachment to the cell wall and protection from fratricins. However, authors have not included any data (eg. cps2A and psr mutants) to demonstrate that these genes do not play a role in providing protection from fratricins. In fact, data from Fig S6 shows increased lysis upon competence stimulation for the Cps2A mutant. This would suggest these players are in fact important to maintain the integrity of the cell during fratricide. It would be beneficial to establish whether these proteins play any role in the phenotypes reported in this manuscript:*

   In figure S6J, the *cps2A* and *psr* mutants are tested for cell lysis in C + Y pH 6.8 media +/- CSP1. Hence, they were tested in the presence of fractricins upregulated during competence and in their absence when competence was not induced. Indeed, the referee is correct that depletion of the capsule makes cells more susceptible to lysis. We have now clarified this in the revised manuscript. Indeed, recent work from the Filipe lab showed that unencapsulated pneumococci are more susceptible to autolysis by LytA and this has now been discussed.

3. *Lines 285-287 and Fig 3C: In contradiction to the statement, the graph (6th panel in Fig 3C) does not show an increased protection (or less lysis) upon LytR overexpression?:*

   Actually, this panel (lytR -/Plac+) should be compared to the first panel (D39V) and it can be observed, especially when 0.25ug/ml CbpD is added, but less when 0.5ug/ml of CbpD was added, that there is additional protection. Most importantly however, both LytR and ComM overexpression concurrently provides better protection to lysis than when each protein is over expressed separately. We have clarified this now in the text to better reflect the data.

4. *Line 312: It may be more accurate to state that ComM is "important" for LytR activity since increased WTA is produced upon LytR induction in a ComM mutant (Table S3):*

   Agreed and we have rephrased this heading.

5. *Lines 324-325: Fig 4B still shows the shift from a fold change of -1.8 to -0.2.*

   Good point. We note that addition of CSP1 would still overproduce LytR in the *comM* mutant thus explaining this partial shift to WTA. This has now been clarified in the revised manuscript.

6. *Fig 4C: Why is there a reduction in WTA/LTA ratio upon overexpression of LytR in a ComM mutant?*

   Again a good point, and we agree this is indeed puzzling and in the revised manuscript we now note that currently we have no explanation for this observation.

7. *Line 214: Can you normalize AUC RLU against the growth? To claim that the low RLU under low IPTG treatment is due to growth defect but not inadequate competence induction in order to rule out the possibility that the gene of interest is indeed regulating the competence development, show that after normalization against growth, there is no significant difference among different IPTG treatment groups.*

   This certainly could be done but does not make the data visualization easier. Any absence of a bar indicates lack of competence induction (e.g. compare *comCDE* NO IPTG to any other plot). This is now better clarified in the figure legend.

8. *Fig. 2D: Shouldn't there also be a condition of lytR-/Plac- with IPTG induction as a complement group of LytR deletion?*

   Complement/ IPTG induction of the *lytR* mutant is shown in Figure 3.

9. *Line 269: In Fig. 3C, the comM overexpression alone (comM-/Plac+) has relieved the lysis compared to ΔcomM or comM-/Plac-. It's likely that ComM alone can deal with CbpD-dependent lysis. You didn't establish that ComM-mediated protection is LytR-dependent by deleting LytR. Suggest testing the condition of comM overexpression in the absence of LytR (comM-/Plac+, lytR-/Plac-). Also, if you compare D39V with lytR-/Plac-, the lysis was even*

worse in the presence of LytR in D39V.

Unfortunately, deleting *lytR* is impossible due to the essential nature of LytR in the D39V genetic background. However, in Fig. 3C we did test for protection from CbpD induced cell lysis in a strain that was depleted for LytR and had normal expression of ComM. As expected, normal expression of ComM, even under low LytR levels, still provides low levels of protection against fratricide.

10. *Fig. 3C: Another issue with Fig. 3C is that it's not convincing to interpret the lysis results with the growth defect. Similar to Point 7 mentioned above, is there a way to normalize the lysis against growth?*

    In principle, the used Y-axis is normalized as suggested by the referee, as we normalize the fluorescence produced by dead cells (484nm emission) over the cell density (595nm). We do this as some depletion strains grow poorly. We have now better clarified this in the figure legend.

11. *Line 370-372: What is the evidence to support this claim that the capsule is thinned by CbpD?*

    The referee is correct that there is no evidence in the literature to support this claim, so we have made it clear in the revised manuscript that this is our current hypothesis.

12. *Line 409: Fig. 5: CbpD-dependent increased surface exposure of PspA and PspC is shown, but not any LytR-dependent effect, which made this part of the story off topic from the detailed analysis of TA. More experiments are needed to show the role of LytR and link to fratricins during this process.*

    Indeed the referee was correct that we did not show any LytR-dependent effect on surface exposure of PspA and PspC. Subsequently, we performed additional fluorescence microscopy experiments, testing LytR depletion and overexpression strains for PspA and PspC surface exposure. These new experiments found that overexpression of LytR amplified the difference in both PspA and PspC surface exposure after competence induction, while low levels of LytR expression abrogated this difference (new Fig. S7).

13. *Line 409: Fig. 5: Data is insufficient to establish causality that the difference in adherence assay (Fig. 5C) is due to different surface exposure of virulence factors. Effects shown in C, E are not shown to be TA dependent. Effects shown in C, E are not shown to explain the 'virulence' effects shown in A. Is adherence and lung superinfection dependent on the display of these CBPs? In general, "competence is crucial for host adherence by increased surface exposure of its various CBPs" is overstated based on the evidence you've shown here.*

    To provide further support for our hypothesis, we now also performed additional nasopharyngeal adherence assays using LytR overexpression and depletion strains. These new experiments showed that indeed overexpression of LytR increased

adherence to nasopharyngeal cells relative to wild type pneumococci, while depletion of LytR reduced adherence levels (new Fig. 3C). These results further support the hypothesis that increased CBP surface exposure due to competence induction impacts adherence to nasopharyngeal cells.

14. *It exactly clear why the LytA-mediated shift from LTA to WTA effects CBPs since both forms of TA are contain phosphocholine? Is the hypothesis that WTA is more exposed than LTA? If so, evidence to support this concept would strengthen the proposed model.*

    This would indeed be in line with our model, but at this point we cannot provide experimental evidence to support this claim other than speculating that it is highly likely that CBPs that are bound to WTA are further away from the membrane than LTA-bound CBPs, and thus likely to be more surface exposed. It is now made more clear in the revision that this is based on speculation and prior assumptions regarding the positions of LTA and WTA within the pneumococcal cell envelope.

Minor Comments:

1. Line 49-51. It is confusing to state that Spn is a commensal and then describe it as a public health problem in the next line.

Text adjusted to clarify potential confusion

2. Line 154 and rest of the manuscript: 1 in CSP1 should not be in subscript.

Adjusted accordingly

3. Line 327: CSP1 and not CPS1

Adjusted accordingly

4. Line 166: not "competent producing competitors", but "competence producing competitors"

Adjusted accordingly

5. Line 167: Give details in the Methods about your cutoff of the top hits.

This information has now been added to the main text 'Fourteen sgRNAs targeting ten operons were significantly less abundant in condition IV, suggesting that they are essential or become more essential in protecting cells against lysis during competence (Figures 1D and S2A, and Table S1; absolute log2FC>1, adjusted p-value<0.05).' and was already mentioned in the Data and code availability section (We tested against a log2FC of 1, with an alpha of 0.05).

6. Line 169: should be "either competence or TA synthesis" but not "both"

Adjusted accordingly

7. Line 185: Even under permissive conditions, a large proportion but not all the cells can become competent?

We and others have shown previously that with the addition of synthetic CSP1, or even during natural competence, all cells in a liquid D39V or R6 population become competent. Citations to these works have now been added (Martin et al., 2010 Mol Micro; Slager et al., 2014 Cell).

8. Line 188: This sentence is ambiguous. "Deletion of comM results in autolysis via CbpD production when competence is activated".

The text now reads "Deletion of *comM* results in autolysis when competence is activated, via CbpD production" to decrease potential ambiguity.

9. Line 194-195: didn't mention what's in grey.

Text edited to explain that in grey, genes involved in neither competence nor teichoic acid biosynthesis are shown.

10. Line 220: reference Fig. S5 instead of Fig. S6

Adjusted accordingly

11. Line 266-267: This sentence is confusing. Do you want to say as LytR is essential for normal growth, so you used lytR-/Plac- instead of a ΔlytR clean deletion to deplete for LytR? Also, in Fig. S6, you added 10μM IPTG to treat Plac-lytR-/+; but you didn't add IPTG to lytR-/Plac- in Fig. 2D. Is that because lytR-/Plac- doesn't require IPTG to remain viable as Plac-lytR-/+ does? The experimental setup is confusing until the reader reaches lines 270-6.

We have now better clarified this in the legend. Indeed, lytR is impossible to delete in the D39V genetic background hence the use of complementation strains. A correct citation has also been added.

12. Line 287: reference Fig. 3C instead of Fig. 3

Adjusted accordingly

13. Line 381: clarify that the 19F strain is EF3030 annotated in Fig. 5

Adjusted accordingly

14. Line 292: In Fig. 3A, should address why the lytR-/Plac- ΔcbpD has more lysis compared to lytR-/Plac-

Indeed, this is an interesting observation as it clearly shows there is less lysis in the exponential phase in the lytR-/Plac- ∆cbpD over the lytR-/Plac- strain, which we attribute to competence-induced CbpD but more lysis in the stationary phase, which we attribute to LytA. A likely explanation for this is that in the absence of some cell wall modification made by CbpD in exponential phase, a larger proportion of cells is susceptible to LytA. Note that this is pure speculation, but we now mention specifically this interesting observation in the revised manuscript.

15. Line 337: reference Fig. 4C instead of Fig. 5C

Adjusted accordingly

16. Line 467: "suggesting their important role in protecting cells"

Adjusted accordingly

17. Line 807: "grown in C+Y medium at pH 6.8"

Adjusted accordingly

18. Line 819: add comma

Adjusted accordingly

19. Line 872: "Calculation of fluorescence intensity based on…"

Adjusted accordingly

20. Line 884: "before adding

Adjusted accordingly (The following day, pneumococcal strains VL1 and VL561 were grown to an OD of 0.1 in C+Y media and 0 ng/ml or 100 ng/ml of CSP1 was added followed by an incubation step of 30 min.)

A second part of the study is the relationship between fratricide and virulence. The data within this part of the story regarding the surface exposure of virulence factors is far too preliminary to support the model that competence promotes adherence to host cells (in vitro) and/or virulence (in vivo) by altered exposure of choline binding proteins on the bacterial cell surface. This reviewer suggests that this aspect of the submission be removed entirely until there is sufficient evidence to support the mechanism the authors are proposing:

Through the addition of new experiments and highlighting more literature, we feel that we have sufficiently linked the relationship between altered exposure of CBPs on the cell surface after competence induction and their link to TA synthesis and virulence/adherence.

We further tested the addition of a LytR depletion and overexpression strains in both the impact on CBP exposure on the cell surface (Fig S7) and in adherence assays on

nasopharyngeal cells (Fig 5C). These new results help to better directly link the relationship between exposure of CBPs on the bacterial surface and virulence. These new results further suggest that TA synthesis impacts deposition of CBPs on the cell surface, and that this impacts pneumococcal ability to adhere to nasopharyngeal cells.

Moreover, we have included additional references into the MS that further highlight the impact of CbpD and CBPs in general on pneumococcal virulence, see below:

The impact of CbpD and CBPs in general on virulence is quite compelling, as it has been shown that the deletion of CbpD inhibits biofilm formation [74], while immunization with purified antibodies against a CbpD peptide fragment significantly increased survival of mice challenged intraperitoneally with lethal levels of a virulent serotype 3 pneumococcal strain [75]. Moreover, the addition of a mutated CSP1 peptide that inhibits the development of competence through outcompeting native CSP1, reduced CbpD and LytA expression levels and significantly decreased murine mortality after pneumococcal intranasal infection [76].
